# Comparison of the virulence of community- and hospital- isolated Acinetobacter baumannii in HeLa cell line and insect model, Galleria mellonella

**DOI:** 10.1099/acmi.0.000858.v3

**Published:** 2025-02-14

**Authors:** Nazmul Hasan Muzahid, Aarthi Ramesh, Tan Hock Siew, Md Zobaer Hasan, Kumaran Narayanan, Sadequr Rahman

**Affiliations:** 1 School of Science, Monash University Malaysia, 47500, Bandar Sunway, Selangor Darul Ehsan, Malaysia; 2 Jeffrey Cheah School of Medicine & Health Sciences, Monash University Malaysia, 47500, Bandar Sunway, Selangor Darul Ehsan, Malaysia

**Keywords:** *Acinetobacter baumannii*, *Galleria mellonella*, HeLa cell line, host-pathogen interaction, pathogenicity

## Abstract

*Acinetobacter baumannii* is an important nosocomial pathogen causing high infections and morbidity among affected individuals, and most studies focus on nosocomial strains. However, *A. baumannii* can also be isolated from healthy community individuals. This study compared the pathogenicity of hospital and community *A. baumannii* isolates using *Galleria mellonella* and human cell cultures. The insect model, *G. mellonella*, and *in vitro* HeLa cell line were used with ten *A. baumannii* isolates (six community and four hospital isolates from Segamat, Malaysia). *G. mellonella* killing assays and HeLa cell adherence, invasion and cytotoxicity assays were performed to investigate the virulence and invasion potential of the isolates. Out of the ten isolates investigated, three community and two hospital isolates were found to be highly virulent in the *G. mellonella* infection model, killing 100% of larvae within 96 h. These strains were also found to be invasive and have significant cytotoxicity in HeLa cells. Our study revealed that community- and hospital-isolated *A. baumannii* could be equally virulent judged by both model systems. Undoubtedly, besides hospital settings, the presence of highly virulent *A. baumannii* in community reservoirs poses a significant public health risk and requires additional investigation.

## Data Summary

Additional data are provided in supplementary materials with the online version of the article.

## Introduction

*Acinetobacter baumannii* is a Gram-negative coccobacillus that is one of the most common causes of hospital-acquired infections, and carbapenem-resistant *A. baumannii* is regarded as the World Health Organization’s top critical priority pathogen for which new therapeutics are urgently needed [1,3]. The bacterium can cause a wide range of illnesses, such as pneumonia, bacteraemia, meningitis and urinary tract infections [4].

*A. baumannii* produces a variety of virulence factors, including lipopolysaccharides, outer membrane proteins and siderophores [5]. These virulence factors allow the bacterium to adhere to host cells, evade immune defences and obtain nutrients. Animal models are frequently used to gain a better understanding of the complex interactions between the microbe and the human host during pathogenesis. Most of the research on *A. baumannii* pathogenesis is performed with murine models [6,7]. However, studies have also been conducted on a wide range of other mammalian species, such as rabbits, guinea pigs and pigs, amongst others [8].

The ethical restrictions and processes involved in using animal models put constraints on the time, repeatability and finances available for *in vivo* experiments. Thus, insect and invertebrate models have been emerging to contribute to *A. baumannii* investigations. Some of these models include, but are not limited to, *Galleria mellonella* (greater wax moth), *Caenorhabditis elegans* (nematode), *Drosophila melanogaster* (fruit fly) and *Danio rerio* (zebrafish) [9]. In particular, huge interest has been shown in*G. mellonella* during the past decade [8]. The use of *G. mellonella* larvae does not need ethical approval, the larvae are inexpensive and simple to maintain at 37 °C using everyday household materials and they have short replication times. Although *G. mellonella* has no adaptive immune response, its cellular and humoral innate immune systems are very well developed [10]. The cellular immune response in larvae is composed of haemocytes, which induce an antimicrobial response by engaging in phagocytosis, encapsulation and clotting [11]. In the *G. mellonella* killing assay, larvae are injected with *A. baumannii*, and their survival is evaluated over time. This assay offers a quick and cost-effective screening method for determining the pathogenicity of isolates.

Aside from *in vivo* models, numerous studies investigating bacterial virulence and pathogenesis have also employed *in vitro* cell line models to gain an initial understanding of the intricate host-pathogen interactions. These studies, which are simpler and more cost-effective than those using *in vivo* models, serve as the foundation for many *in vivo* studies by providing additional data to support any conclusions [12]. Epithelial mucous membrane cells are the primary focus of most *in vitro* investigations due to them being usually the initial point of contact for infections [12,13]. HeLa cells, which originate from human cervical epithelial cells, are thus frequently selected for bacterial adhesion and invasion and are particularly suitable for experiments [14]. *A. baumannii* frequently infects human epithelial tissues, such as the respiratory system, skin and mucosal linings [15]. HeLa cells are resilient and readily cultured *in vitro*, exhibiting a rapid growth rate. This ensures the availability of a uniform and consistent cell population for studies, rendering them economical and reliable.

In this study, we compared the pathogenicity of hospital- and community-isolated *A. baumannii* in insect model *G. mellonella* and *in vitro* HeLa cell line. We hypothesized that hospital-isolated strains are more virulent than community isolates. This study aims to investigate the effects of community- and hospital-isolated *A. baumannii* infections in simple host models and rank the isolates in terms of pathogenicity.

## Methods

### Bacterial strains and culture

All *A. baumannii* strains used in this study were collected throughout the course of a community research project that involved the isolation and investigation of antibiotic-resistant and virulent pathogens from healthy community individuals and co-located hospitals in the Segamat district, state of Johor, Malaysia. In the previous study, we reported the molecular characterization, antimicrobial susceptibility profile and comparative whole-genome sequencing of *A. baumannii* isolates [16]. In the current study, ten *A. baumannii* strains were used, with six isolated from the community and four from the hospital. The community strains were isolated from faecal samples, and hospital isolates were obtained from hospitalized patients. Details of the isolates are attached in Tables S1 and S2 (available in the online Supplementary Material). All ten *A. baumannii* strains used in this study were susceptible to antibiotic gentamicin.

### HeLa cell culture

The human cervical carcinoma (HeLa) cell line was cultured in Dulbecco’s modified Eagle’s medium (DMEM) (Gibco) supplemented with 10% FBS (Gibco; Thermo Fisher Scientific, Inc.). The cells were maintained at 37 °C in a 5% CO_2_ incubator. Cells were then seeded at a density of 5×10^4^ and 2.5×10^4^ in 12- and 24-well plates for the adherence, invasion and cytotoxicity assays, respectively (Nunclon™–Nalgene Nunc International, Rochester, NY).

### Virulence assay using the *G. mellonella* infection model

An *in vivo* infection assay was conducted on the larger wax moth, *G. mellonella*, to ascertain the virulence of *A. baumannii*. The infection experiments were performed as previously described in [17]. Briefly, triplicate assays of ten larvae (250–300 mg) were injected with 1×10^7^ and 1×10^6^ in *A. baumannii* isolates. To eliminate the chance of bias, larvae that showed symptoms of melanization or deformation were taken out of the sample. As a negative control, 10 µl of PBS was injected into one group of larvae. This was done to see if the larvae died because of physical damage. Another group that was used as a control did not get an injection. The larvae were kept in an incubator for 7 days at 37 °C. Every 24 h, signs of death were looked for. Larvae that did not move when touched or underwent a black colouration were identified as dead. Larval health and survival were monitored every day post-injection for 7 days.

### Bacterial adherence assay in human host HeLa cells

Adherence assay of *A. baumannii* isolates in HeLa cells was performed as described previously with some modifications [18]. Strains of *A. baumannii* were cultured in 10 ml LB broth at 37 °C overnight. Bacterial suspensions were washed and adjusted to match the turbidity standard of one McFarland unit (~3.0×10^8^ c.f.u. ml^−1^) in plain DMEM without FBS. The adherence assays were conducted by exposing the HeLa cells at a multiplicity of infection (MOI) (bacterium: eukaryotic cell ratio) ~2000 : 1 of *A. baumannii* isolates. The infected plates were centrifuged for 10 min at 300 ***g*** prior to incubation to promote bacterial adhesion to cells and synchronize infections. Each strain was tested in triplicate. After 2 h of incubation at 37 °C, 5% CO_2_, coverslips were washed five times with 1X analytical grade sterile PBS, fixed with 100% ice-cold methanol by rocking for 15–20 min. The coverslips were washed twice with 1X analytical grade sterile PBS and stained with 2% Giemsa solution for 30 min. After washing thrice with 1X analytical grade sterile PBS, the coverslips were mounted on glass slides using a mounting solution. *A. baumannii* adhesion to cells was determined by optical microscopy.

### Bacterial invasion assay

HeLa cells seeded in 24-well plates were washed thrice gently with 1X analytical grade sterile PBS. *A. baumannii* suspensions prepared in sterile plain DMEM (without FBS) were added into the wells with an MOI 1 : 2000 (bacterium: eukaryotic cell ratio ~2000 : 1). Plates were centrifuged at 300 ***g*** for 10 min at room temperature in a swing bucket centrifuge machine. Plates were then incubated in a human cell incubator at 37 °C, 5% CO_2_ for 2 h. After incubation, cells were washed thrice gently with 1X analytical grade PBS. Antibiotics were now added to kill extracellular bacteria in two intervals. First, 80 µg ml^−1^ of gentamicin was added to each well in sterile plain DMEM (without FBS) and incubated for 2 h in the human cell incubator at 37 °C, 5% CO_2_. After 2 h, cells were washed twice gently with 1X analytical grade PBS (sterile and autoclaved). Now, 20 µg of gentamicin was added to each well in sterile plain DMEM (without FBS) and incubated for 48 h in the human cell incubator at 37 °C, 5% CO_2_. After the long incubation, existing media was removed, and cells were washed gently five times with 1X analytical grade PBS. One millilitre of 0.1% Triton X-100 lysis buffer was added to all wells and incubated for 20 min at 37 °C. After vigorous resuspension, the plates were then gently shaken, and the contents were immediately transferred to a microcentrifuge tube. Plating was done after two serial dilutions. One hundred microlitres of the suspension were used for plating and incubated for 24 h in a bacterial cell incubator at 37 °C to observe and count colonies. Colonies were then counted and analysed to determine *A. baumannii* invasiveness. We used the bacterial invasive strain *Escherichia coli* DH10β asd- (pGB2Ωinv-hly, pEGFPN2) as a positive control for bacterial invasion and non-invasive *E. coli* DH5α as a negative control [14].

### Trypan blue cell cytotoxicity assay

Plates were washed thrice with 1X PBS, and *A. baumannii* suspensions were added into the HeLa cell culture (MOI, bacterium: eukaryotic cell ratio ~2000 : 1). Plates were centrifuged at 300 ***g*** for 10 min at room temperature. Plates were incubated for 2 h at 37 °C in a 5% CO_2_ incubator. Plates were washed thrice with 1X PBS. Cells were trypsinized with 300 µl of 0.25% Trypsin EDTA. Three hundred microlitres of complete media were then added to quench the activity of trypsin. Cell suspension was centrifuged at 1000 r.p.m. for 5 min at room temperature. Two hundred microlitres of PBS were then added and centrifuged again at 1000 r.p.m. for 5 min. The supernatant was discarded, and the pellet was resuspended with 250 µl PBS. The microcentrifuge tubes were kept in a styrofoam box to avoid cell death until the trypan blue assay was complete. A 1 : 1 ratio of cell mixture and trypan blue (10 µl each) was used. From the 20 µl mixture, 10 µl was taken into the haemocytometer for counting.

### Statistical data analysis

All *Galleria* killing assay and HeLa cell analyses were performed by three replicates using GraphPad Prism software 10.2 (La Jolla, CA, USA). For the killing of *G. mellonella* by the hospital- and community-isolated *A. baumannii*, survival curves were graphed and analysed by log-rank (Kaplan–Meier) test using the GraphPad Prism 10.2 software, and median survival times were obtained for each strain. The significance of differences for the *A. baumannii* invasion of HeLa cells and a two-tailed t-test was applied to estimate the mean±sd, and cellular cytotoxicity was determined using ANOVA and Tukey’s post hoc tests. The correlation between the *Galleria* killing assay and human cell invasion assay was carried out using a normal distribution (Q-Q plot), when the requirements were not met, then performed ‘Spearman correlation’ and ‘Mann-Whitney test’ by using SPSS v21 (IBM Corp., Armonk, NY, USA). *P*-values of ≤ 0.05 were considered significant.

## Results

### Community and hospital isolates were significantly virulent in *G. mellonella* killing assay

**Fig. 1.**
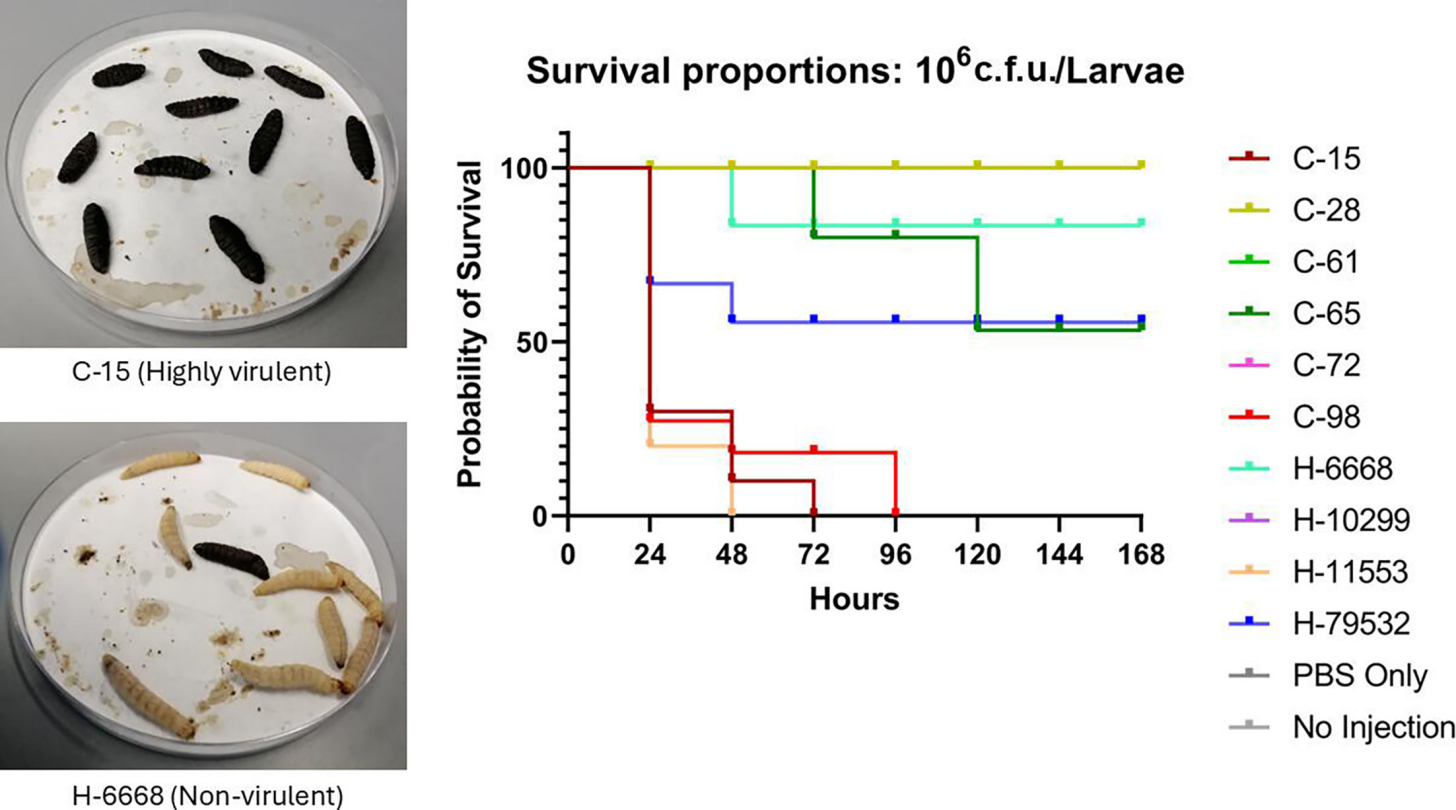
Kaplan–Meier log-rank survival distributions and median survival times for dose-dependent challenges of *A. baumannii isolates* in ten *G*. *mellonella* larvae. Here, 10^6^ c.f.u./larva were injected for each strain. Injection of PBS only and no injection were used as controls. The results, presented as a percentage chance of survival, are based on a pool of three biological replicates for each experiment. Infection results for all ten isolates tested were significant (*P*=0.001; Mantel–Cox log-rank test) compared to the 'no injection' control, demonstrating that larval survival depends on the number of bacteria injected. In addition, statistically significant differences between infections by different bacterial strains were also noted.

*G. mellonella* larvae serve as an animal model and are useful for determining the pathogenicity of micro-organisms, including *A. baumannii*. Therefore, we employed this model to study the pathogenicity of 10 community- and hospital-isolated *A. baumannii* strains. Fig. 1 highlights the variability of pathogenicity at 10^6^ c.f.u./larva concentrations among different strains.

It was found that parenteral injection of 10^6^ and 10^7^ c.f.u. of *A. baumannii* strains led to the death of *G. mellonella* larvae. Results of 10^7^ c.f.u./larva are shown in the supplementary file (Fig. S1). Infected larvae showed nodulation, cuticle blackening and ultimately death. The frequency of melanization in larvae increased strikingly with increasing inoculum doses, suggesting that the initial infectious inoculum is vital for developing the infection. The Kaplan–Meier survival distributions for each bacterial inoculum were significantly different when compared using the log-rank (Mantel–Cox) test (*P*<0.001) and the survival probability was dependent upon the number of organisms injected (Figs 1 and S1). Larvae injected with an inoculum size of 10^6^ c.f.u./larva exhibited 90–100% survival after 168 h for two community strains (C-28 and C-61) and one hospital strain (H-6668). Furthermore, 60 and 8o% survival rates were detected for one hospital strain (H-79532) and one community strain (C-65), respectively. Our data suggested the lesser virulent nature of these three community and two hospital strains. On the other hand, three community strains (C-15, C-72 and C-98) and two hospital strains (H-10299 and H-11553) killed all the larvae within 48–72 h (Fig. 1). We used another inoculum size of 10^7^ c.f.u./larva to compare the virulent nature and found considerable differences (Fig. S1). We found a 0% survival rate after 24 h for two community strains (C-15 and C-98) and two hospital strains (H-10299 and H-11553), and after 48 and 72 h, we found a 0% survival rate for another community (C-72) and two hospital isolates (H-6668, H-79532), respectively (Fig. S1). Within the 7-day time point (168 h), 100% of larvae were killed by the remaining three *A. baumannii* strains, indicating that the virulence of all these strains is dose-dependent.

### HeLa cell adherence assay

All ten *A. baumannii* isolates, representing five highly virulent and five non-virulent isolates as determined by the *Galleria* assay, were used for further analysis. The adherence of these ten isolates to HeLa cells was determined. From the adherence assay, we found that all ten *A*. *baumannii* strains were adherent to HeLa cells (Fig. S2). However, virulent strains identified in the *G. mellonella* infection assay (C-15, C-72, C-98 and H-11553) showed morphological disturbance in cells resembling cell death (Fig. S2). Our adherence assay data indicates that all *A. baumannii* strains investigated showed adherence potential; however, only virulent strains caused cellular damage. It is observed that *A. baumannii* strains identified as highly virulent from *G. mellonella* killing assay (e.g. C-98) showed less adherent potential as compared to strains identified as less virulent (e.g. C-61). This could suggest that highly invasive/virulent strains enter cells more rapidly than less/non-invasive strains and are thus less visible as being adherent. This adhesion difference between virulent and non-virulent strains could be a factor for future investigation.

### HeLa cell invasion assay

**Fig. 2.**
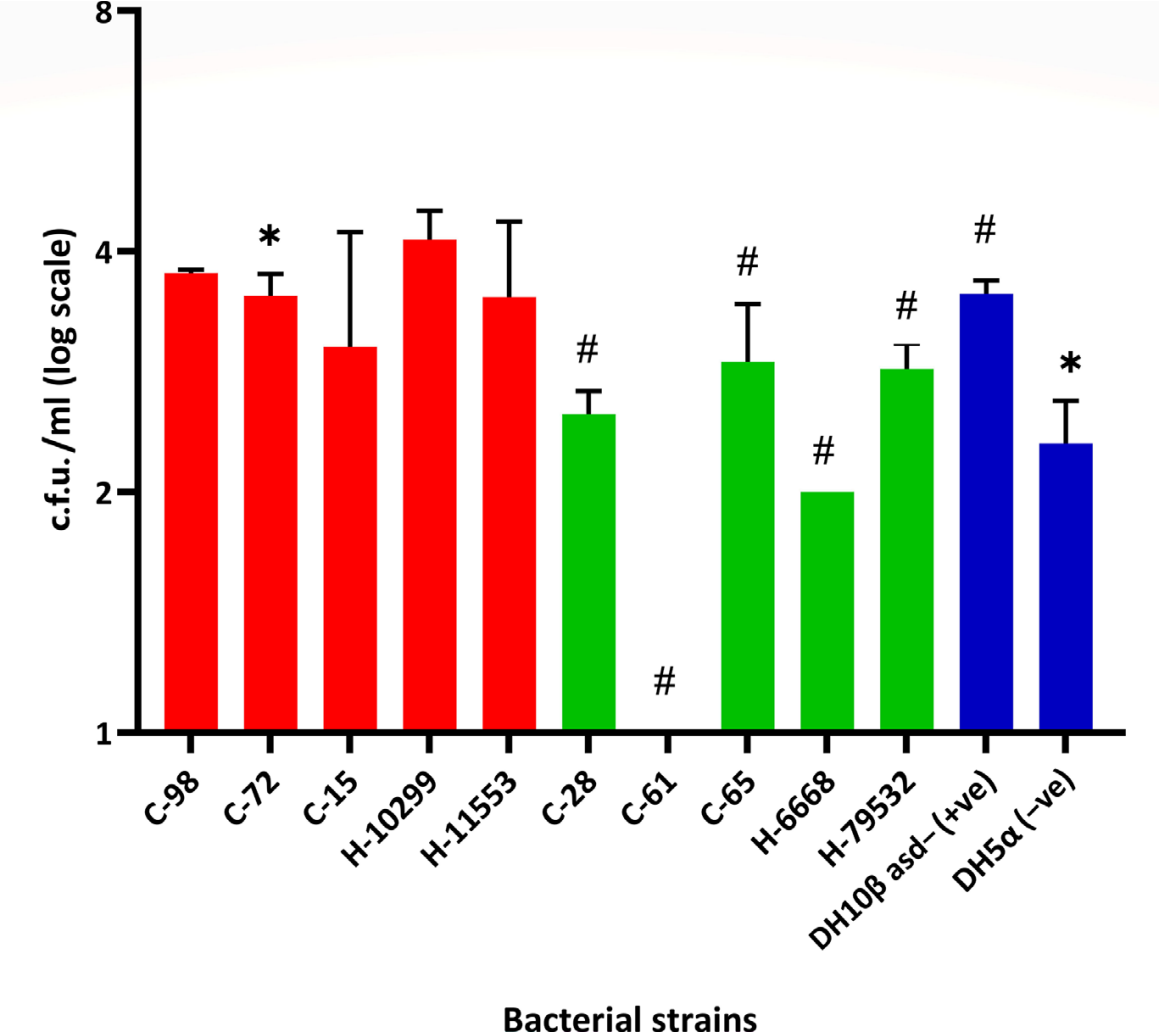
Number of internalized *A. baumannii* strains after invasion into the HeLa cell line at an MOI of 1 : 2000 (mammalian cell : bacterial cell ratio) for 2 h. Invasion efficiency was expressed as the number of bacteria per well using the gentamicin protection assay. The results were presented as the mean±sd c.f.u. ml^−1^ of three independent repeats and compared to *E. coli* strain DH10β asd- as positive control and DH5α as negative control. Asterisk (*) and hash (^#^) represent statistical significance (*P* ≤ 0.05) against positive and negative control, respectively, using a two-tailed t-test. All five non-virulent strains (C-28, C-61, C-65, H-6668 and H-79532) showed significant differences against positive control, whereas only virulent strain C-72 showed significant differences against the negative control. Here, red bars indicate that those five isolates were pathogenic in the *Galleria* infection model, whether the remaining five isolates with green bars were non-pathogenic. Blue bars are the control strains.

From the adherence assay, we found that *A. baumannii* strains can adhere to the HeLa cells, effectively indicating their potential for invasion. Interestingly, we found the less adherent ones as being the ones that caused greater distortion of cell shape, suggesting that they were more pathogenic. We used the same ten *A*. *baumannii* community and hospital strains as invaders against the HeLa host cell line. Besides, *E. coli* DH10β asd- (pGB2Ωinv-hly, pEGFPN2) and *E. coli* DH5α were used as positive and negative controls for bacterial invasion, respectively. The invasion assay showed that the community strains C-98 and C-72 and hospital strains H-10299 and H-11553 showed high invasiveness with >800 c.f.u. ml^−1^ (Fig. 2). The invasiveness of these strains in HeLa cells also correlated with their killing potential in the *G. mellonella* killing assay. This study shows that community-isolated *A. baumannii* are as pathogenic as hospital isolates, thus indicating that more caution is required when in contact with these strains.

### Trypan blue cytotoxicity assay

**Fig. 3.**
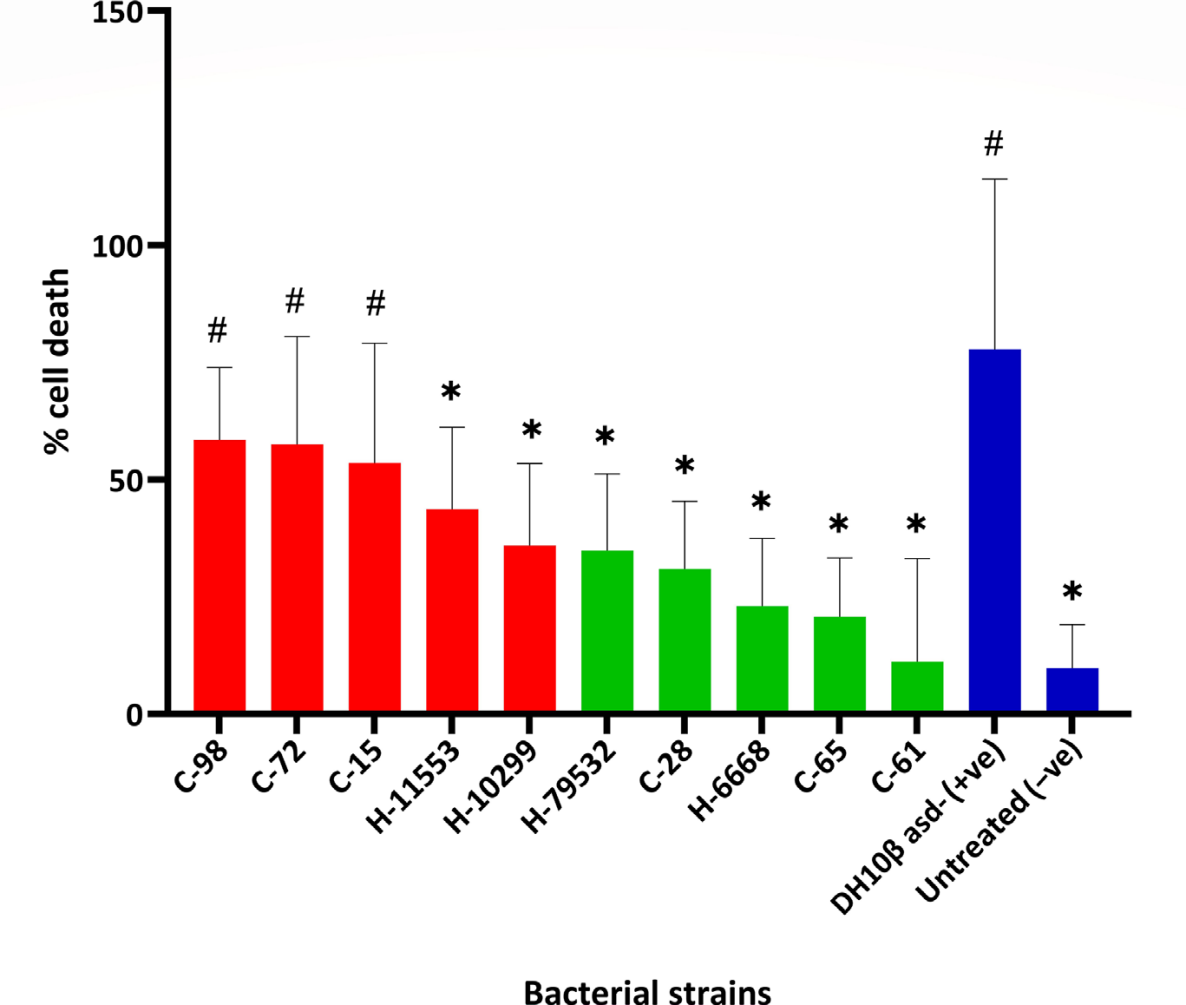
Cellular cytotoxicity of *A. baumannii* strains presented as % cell death in the host HeLa cell line. Here, red bars indicate that those five isolates were pathogenic in the *Galleria* infection model, whether the remaining five isolates with green bars were non-pathogenic. Blue bars are the control strains. *E. coli* strain DH10β asd- was used as a positive control, and untreated cells were used as negative controls. Asterisk (*) and hash (^#^) represent statistical significance (*P*≤0.05) against positive and negative control, respectively, using ANOVA.

After determining bacterial invasiveness in the HeLa cell line, we aimed to determine their effects on cellular cytotoxicity. We used trypan blue assay 48 h post-treatment with gentamicin for both community- and hospital-isolated *A. baumannii* strains to do this. We found a significant increase in % cell death by virulent *A. baumannii* strains from the community (C-98, C-72 and C-15). Although hospital invasive/virulent strains (H-10299 and H-11553) identified from *G. mellonella* killing and HeLa cell invasion assays did not show statistical significance compared to the positive control, we still observed a higher % cell death compared to other non-virulent strains (Fig. 3). Cells infected with ten *A*. *baumannii* isolates, along with positive and untreated negative controls, showed consistent cytotoxicity outcomes, which correlated well with their human host cell invasion assay and *G. mellonella* killing assay outcomes. The results show that these virulent strains (C-98, C-72, C-15, H-10299 and H-11553) are not only invasive but also cytotoxic to human cells. The cytotoxicity outcomes become more important as there is preliminary evidence that these bacterial strains may cause harm to infected individuals in the future.

### Comparison of the pathogenicity of both hospital and community strains

Using two independent virulence measures, we find that some of the community strains isolated in this study are as pathogenic as those isolated from the co-located hospital. We cannot distinguish them based on virulence using our small sample size. Statistically significant negative correlations were found between the survival time of *Galleria* larvae infected with indicated strains and their HeLa cell invasion (Tables S6−S8). Details of the statistical treatment are available in the supplementary material.

## Discussion

*A. baumannii* is a pathogen of great concern. Although it is normally isolated from hospital wards, we find that about 3% of the general community produced faeces that allowed the isolation of *A. baumannii* isolates [16]. Using two model systems, this study shows that community isolates are potentially pathogenic, similar to those isolated from the co-located hospital. Earlier, we had shown that although community strains were less likely to be resistant to commonly used antibiotics, some strains were closely related to hospital isolates [16]. Those results and the virulence results reported here indicate the importance of reducing the transmission of *A. baumannii* between hospital and community settings. Some community strains could lead to life-threatening infections if the host immunity is compromised.

Although mammalian models remain the most effective for studying host-pathogen interaction, interest in non-mammalian models has increased due to their benefits over mammalian equivalents. Non-mammalian models are not limited by ethical constraints and are more cost-effective than mammalian models, as they do not require expensive keeping facilities. These variables enable researchers to use a large number of organisms in a study, making it easier to do high-throughput screening for bacterial virulence factors, toxins and overall bacterial pathogenicity. These studies can use a wide variety of hosts and bacterial strains to quickly analyse emerging clinical strains [12].

Even with a small sample size, our study unveiled the virulence and pathogenicity of community *A. baumannii* strains, which were as significantly infectious as the hospital strains. *G. mellonella* has been used in recent years to describe the virulence traits of numerous pathogens, including *A. baumannii*, *Pseudomonas aeruginosa*, *Burkholderia cepacia*, *Bacillus cereus* and several disease-causing fungi [19,20]. Due to its endurance of incubation temperatures up to 37 °C, *G. mellonella* is more suitable for studying human infection compared to other model systems like *C. elegans* [20]. We used *Galleria* to investigate and compare the virulence of *A. baumannii* isolated from the community and hospital (Fig. 1). Higher inoculum (10^7^ c.f.u./µl) from most isolates killed more promptly compared to lower bacterial inoculum (10^6^ c.f.u./µl), indicating that *G. mellonella* is susceptible to *A. baumannii* infection in a dose-dependent manner (Fig. 1).

A variety of factors contribute to *A. baumannii* virulence and pathogenicity. Previous studies have demonstrated that *A. baumannii* isolates exhibit a wide variety of virulence features [21,22]. However, little is known about *A. baumannii* adherence and invasion in human cells. Interestingly, from the HeLa cell adherence assay, we found that *A. baumannii* strains, which were found to be highly virulent from *G. mellonella* killing and human host invasion assays (e.g. C-98), showed less adhesion to HeLa cells as compared to less virulent strains (e.g. C-61). Several studies suggest that adhesion is important for bacterial infections as it mainly helps avoid bacterial clearance by host mucosal secretions and peristalsis [23]. Adhesion and stickiness of the bacteria have been identified to aid in its invasiveness and virulence [24]. However, our study found that the strains with less virulence potential showed more adhesion on and outside the host human cells. This adhesion potential of these less virulent strains did not translate into higher invasiveness and cytotoxic potential (Fig. 3). Thus, the adhesion potential of these invasive and virulent strains could clearly be an important objective of our future investigation. We discovered that a total of five isolates were highly virulent where three of them were isolated from the community (C-98, C-72 and C-15) and two from the hospital (H-10299 and H-11553) (Tables S3–S5 for their rank order for statistical analysis). Virulent *A. baumannii* isolated from community individuals is of great concern.

Our study revealed that the *in vivo Galleria* infection model and *in vitro* cell culture could be used as a routine way to detect the pathogenicity of *A. baumannii*. The significant statistical correlations between the two tests suggest that either or both models could be used as a preliminary screen before selecting strains for detailed *in vivo* assessment in a mammalian model system.

A significant strength of this study is the use of complementary model systems, which lend both *in vivo* and *in vitro* perspectives on bacterial virulence. The *G. mellonella* model provides a cheaper, high-throughput way of assessing pathogenicity in controlled environments, and the HeLa assays give insight into the interactions of bacteria with human cell lines. This dual methodology improves the reliability of the results and facilitates future research. Additionally, the identification of virulent community strains emphasizes an urgent public health issue and will enhance our understanding of the relationship between hospital and community isolates.

However, there are limitations to this study. The number of isolates assayed was small, and this may limit the generalizability of results. These findings will need to be replicated in larger, geographically distributed datasets. Moreover, although * G. mellonella* is an adequate initial model, it does not fully recapitulate the diversity of mammalian immune responses, and studies in mammalian systems will be necessary to continue in-depth investigations. Finally, although adhesion assays provided valuable insights, the exact molecular mechanisms underlying the differences in adhesion and virulence remain unclear and warrant further investigation.

## Conclusion

Determination of the virulence or pathogenicity of bacterial isolates is of critical importance for public health. However*, in vivo* studies of isolates are expensive and time-consuming. As a result, despite the importance, most studies of *A. baumannii* pathogenicity have been conducted on isolates from hospital sources. In contrast, little attention has been paid to the community reservoir of *A. baumannii* strains. Our study has revealed that the community isolates of *A. baumannii* are as virulent and pathogenic as hospital isolates. Both *G. mellonella* and human cell line assays give us highly correlated data to confirm the potential virulent nature of our *A. baumannii* strains. Future studies could be conducted using a large sample size of *A. baumannii* isolates to determine the threat to public health from the community reservoir.

## supplementary material

10.1099/acmi.0.000858.v3 Uncited Fig. S1.

## Abbreviations

c.f.u.: colony forming unit
DMEM: Dulbecco’s modified Eagle’s medium
FBS: fetal bovine serum
MOI: multiplicity of infection
PBS: phosphate buffered saline
