# Supplementary material for: Comparison of the virulence of community- and hospital- isolated Acinetobacter baumannii in HeLa cell line and insect model, Galleria mellonella
Source: Access Microbiol. 2025 Feb 14;7(2):000858.v3. doi: 10.1099/acmi.0.000858.v3 (PMC11829074; doi:10.1099/acmi.0.000858.v3)
Supplement: Uncited Fig. S1. [file acmi-7-00858-s001.pdf]

Supplementary Material

Comparison of the virulence of community and hospital isolated *Acinetobacter baumannii* in HeLa cell line and the insect model, *Galleria mellonella*

Nazmul Hasan Muzahid[1], Aarthi Ramesh[1], Tan Hock Siew[1], Md Zobaer Hasan[1], Kumaran Narayanan[2], Sadequr Rahman[1]*

[1]School of Science, Monash University Malaysia, 47500, Bandar Sunway, Selangor Darul Ehsan, Malaysia

[2]Jeffrey Cheah School of Medicine & Health Sciences, Monash University Malaysia, 47500, Bandar Sunway, Selangor Darul Ehsan, Malaysia

***Corresponding authors**:

Nazmul Hasan Muzahid; nazmul.muzahid@monash.edu

Sadequr Rahman; sadequr.rahman@monash.edu

TABLE S1: Details of *A. baumannii* isolates from the community in Segamat, Malaysia

SL no.	Sample Collection Year	Strain ID	Location	Relationship	Gender	Age	Ethnicity	Isolation source
1	2018	C-15	Chaah	Head	Male	62	Indian	Feces
2	2018	C-28	Chaah	Wife	Female	50	Indian	Feces
3	2018	C-61	Bekok	Head	Male	39	Chinese	Feces
4	2018	C-65	Chaah	Head	Male	69	Indian	Feces
5	2018	C-72	Chaah	Head	Female	54	Indian	Feces
6	2018	C-98	Chaah	Wife	Female	46	Indian	Feces

TABLE S2: Details of *A. baumannii* isolates from the Segamat hospital, Malaysia

SL No.	Strain ID	Sample Collection Year	Patient Age	Gender	Diagnosis	Isolation source
1	H-6668	2020	63	Male	Community acquired pneumonia	Blood
2	H-10299	2020	79	Male	Septic shock	Tracheal aspirate
3	H-11553	2020	75	Male	Community acquired pneumonia	Tracheal aspirate
4	H-79532	2020	28	Male	Traumatic Brain Injury	Tracheal aspirate

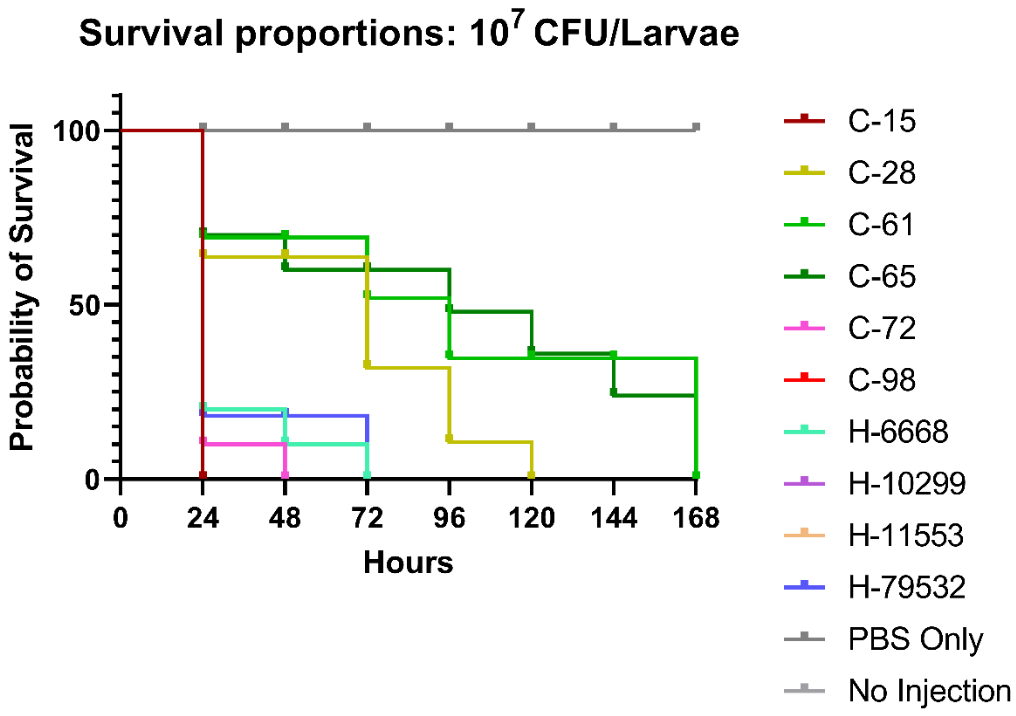

Fig. S1: **Kaplan-Meier Log-Rank survival distributions and median survival times for dose-dependent challenges of *A. baumannii* isolates in 10 *G. mellonella* larvae.** Here, 10^7 CFU/larvae were injected for each strain. Injection of phosphate buffer saline (PBS) only and no injection were used as a control. The results, presented as a percentage chance of survival, are based on a pool of three biological replicates for each experiment. Infection results for all ten isolates tested were significant ($p = 0.001$; Mantel-Cox log rank test) demonstrating that larval survival is dependent on the quantity of bacteria injected.

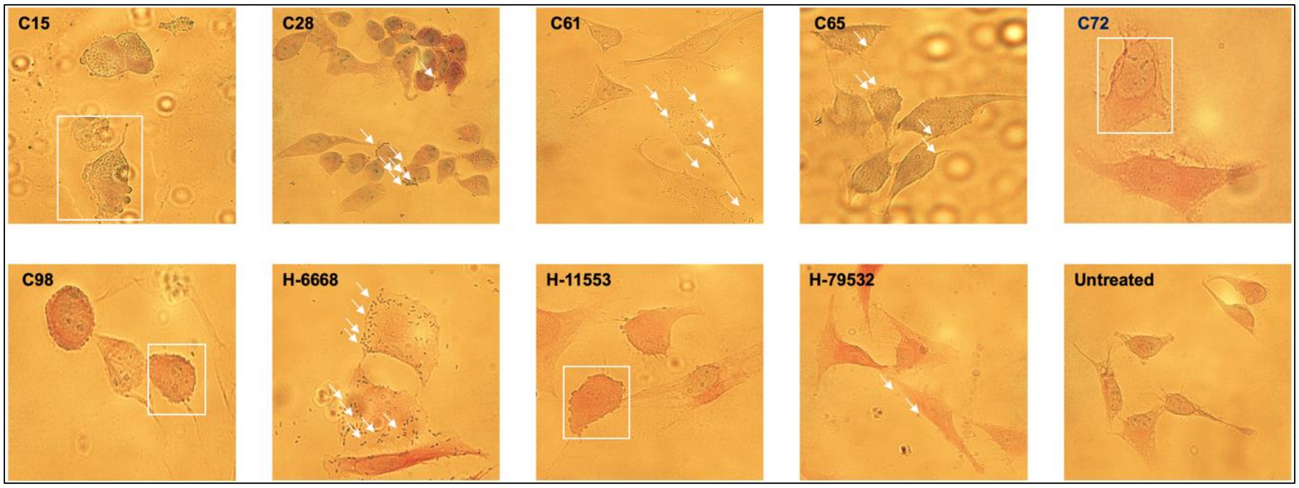

Fig. S2: HeLa cells infected with *A. baumannii* isolates (C-15, C-28, C-61, C-65, C-72, C-98, H-6668, H-11553, H-79532) for 2 hours, stained with 2% Giemsa solution and photographed under an optical microscope at a 40x magnification. Here, all 10 *A. baumannii* strains were adherent to HeLa cells. However. only virulent strains (C-15, C-72, C-98, H-11553) caused cellular damage (white square). White arrows showing non-virulent strains adhere to the cells.

TABLE S3: Insect Data: 10^6 CFU/ml

	R1				R2				R3				Average	
**Isolates**	**Total number killed**	**Hours to killed**	**Average time taken to kill 1**	**Rank**	**Total number killed**	**Hours to killed**	**Average time taken to kill 1**	**Rank**	**Total number killed**	**Hours to killed**	**Average time taken to kill 1**	**Rank**	**Rank Score**	**Rank**
**H-11553**	10	48	4.8	1	10	48	4.8	1	10	48	4.8	1	1	1
**H-10299**	10	72	7.2	2	10	72	7.2	2	10	72	7.2	2	2	4
**C-15**	10	72	7.2	2	10	72	7.2	2	10	48	4.8	1	1.666666667	3
**C-72**	10	48	4.8	1	10	48	4.8	1	10	72	7.2	2	1.333333333	2
**C-98**	10	48	4.8	1	10	48	4.8	1	10	96	9.6	4	2	4
**H-79532**	2	48	24	3	4	48	12	3	4	24	6	3	3	5
**C-65**	6	144	24	3	1	120	120	5	2	168	84	5	4.333333333	6
**H-6668**	1	48	48	4	1	48	48	4	1	240	240	6	4.666666667	7
**C-28**	1	240	240	6	1	240	240	6	1	240	240	6	6	9
**C-61**	1	168	168	5	1	240	240	6	1	240	240	6	5.666666667	8

TABLE S4: Human HeLa cell invasion data (Number of bacteria invaded)

Isolates	R1	Rank	R2	Rank	R3	Rank	Average Rank Score	Rank
**H-11553**	8	4	300	1	14	5	3.333333333	2
**H-10299**	300	1	147	2	58	3	2	1
**C-15**	6	5	1	7	218	1	4.333333333	4
**C-72**	43	2	18	5	47	4	3.666666667	3
**C-98**	0	9	54	3	61	2	4.666666667	5

**H-79532**	10	3	4	6	9	6	5	6
**C-65**	2	7	19	4	14	5	5.333333333	7
**H-6668**	1	8	0	8	1	8	8	9
**C-28**	4	6	4	6	2	7	6.333333333	8
**C-61**	0	9	0	8	0	9	8.666666667	10

TABLE S5*:* *A. baumannii* invasion of HeLa cell line data CFU/ml

	**C-98**	**C-72**	**C-15**	**H-10299**	**H-11553**	**C-28**	**C-61**	**C-65**	**H-6668**	**H-79532**	**DH5α (-ve control)**	**DH10β, ASD- (+ve control)**
Replicate_1	0	4300	600	30000	800	400	0	200	100	1000	100	2900
Replicate_2	5400	1800	100	14700	30000	400	0	1900	0	400	200	5000
Replicate_3	6100	4700	21800	5800	1400	200	0	1400	100	900	400	2900
STD	3338.163	1571.623	12386.69	12240.23	16688.12	115.4701	0	873.6895	57.73503	321.4550254	152.7525232	1212.435565
AVG	3833.333	3600	7500	16833.33	10733.33	333.3333	0	1166.667	66.66667	766.6666667	233.3333333	3600

Statistically significant negative correlations were found between the time of survival of Galleria larvae infected with indicated strains and their HeLa cell invasion

During infection in the Galleria killing assay, all the insects died within seven days timepoint at 10^7 bacterial loads. However, we found differences in bacterial virulence at 106 concentrations; thus, we used it for the statistical analysis. In our Q-Q plot analysis, the raw data in the Galleria insect model **(total killing time)** and HeLa cell invasion assay **(number of invading bacteria)** do not follow the normal distribution. That's why we run the Spearman Correlation to investigate the correlation between the "raw Insect Data and raw Human Data". We revealed **statistically significant negative correlation** (the bacteria which take less time to kill a certain number of insects are invading more human cells in some certain time) between the insect (time to kill Galleria) and human cell invasion (number of invading bacteria) data (Table S6). In addition, the Spearman correlation between the "rank of Galleria insect data & human cell invasion data" showed a significant positive correlation (the bacteria that get some high ranking by killing ability in the insect data, the same bacteria getting high ranking in the HeLa cell invasion data) (Table S7).

TABLE S6: Spearman correlation between the raw Galleria Insect Data and human HeLa cell invasion Data (Output 3)

		**Nonparametric Correlations**	AVG_Galleria_killing_Rank	AVG_HeLa_cell_Invasion_Rank	R1_Rank	REP1_Rank	R2_Rank	REP2_Rank	R3_Rank	REP3_Rank
Spearman's rho	AVG_Galleria killing_Rank	Correlation Coefficient	1.000	-.790**	1.000	-.372	1.000	-.621	1.000	-.660*
		Sig. (2-tailed)	.	.007	.	.290	.	.055	.	.038
	AVG_HeLa cell_invasion_Rank	Correlation Coefficient	-.790**	1.000	-.372	1.000	-.621	1.000	-.660*	1.000
		Sig. (2-tailed)	.007	.	.290	.	.055	.	.038	.

Here, R1, R2, R3 Rank are the three replications of the Galleria Insect killing assay Rank. REP1, REP2, REP3 Rank are the three replications of the HeLa cell invasion Rank. **Highly significant negative correlations was found.**

TABLE S7: Spearman correlation between the rank of Galleria insect data and HeLa cell invasion Data

		Nonparametric Correlations	AVG_Galleria_killing_Rank	AVG_Hela_cell_invasion_Rank	R1_Rank	REP1_Rank	R2_Rank	REP2_Rank	R3_Rank	REP3_Rank
Spearman's rho	AVG_Galleria_killing_Rank	Correlation Coefficient	1.000	.869**	1.000	.372	1.000	.621	1.000	.746
		Sig. (2-tailed)	.	.001		.290		.055		.013
	AVG_HeLa_cell_Invasion_Rank	Correlation Coefficient	.869**	1.000	.372	1.000	.621	1.000	.746*	1.000
		Sig. (2-tailed)	.001	.	.290		.055		.013	

Here, R1, R2, R3 Rank are the three replications of the Galleria Insect killing assay Rank. REP1, REP2, REP3 Rank are the three replications of the HeLa cell invasion Rank. **Highly significant positive correlations was found.**

We also used the Mann-Whitney Test to test the hypotheses using $\alpha = 0.05$: Here, H_0: The distribution of the average time taken to kill one insect by a bacterium and the distribution of average HeLa cells invaded by the same bacteria are identical. H_a: The distribution of the average time taken to kill one insect by a bacterium and the distribution of average HeLa cells invaded by the same bacteria are not identical.

TABLE S8: Mann-Whitney Test between the average and replicates of Galleria Insect killing Data & human HeLa cell invasion Data

	Average	Replication 1	Replication 2	Replication 3
Mann-Whitney U	43.000	30.000	37.000	42.000
p-value	0.597	0.130	0.324	0.544

Since the p-values are greater than the $\alpha = 0.05$ for all the cases (i.e., average and three replicates), therefore, we can't reject H_0 and conclude that the distribution of the average time taken to kill one insect by a bacterium and the distribution of average HeLa cells invaded by the same bacteria are identical (Table S8).
